# Supplementary material for: Molecular Identification of African Nymphaea Species (Water Lily) Based on ITS, trnT-trnF and rpl16
Source: Plants (Basel). 2022 Sep 18;11(18):2431. doi: 10.3390/plants11182431 (PMC9503883; doi:10.3390/plants11182431)
Supplement: Supplementary file 1 [file plants-11-02431-s001.zip › Supplementary Figures S1-S7.pdf]

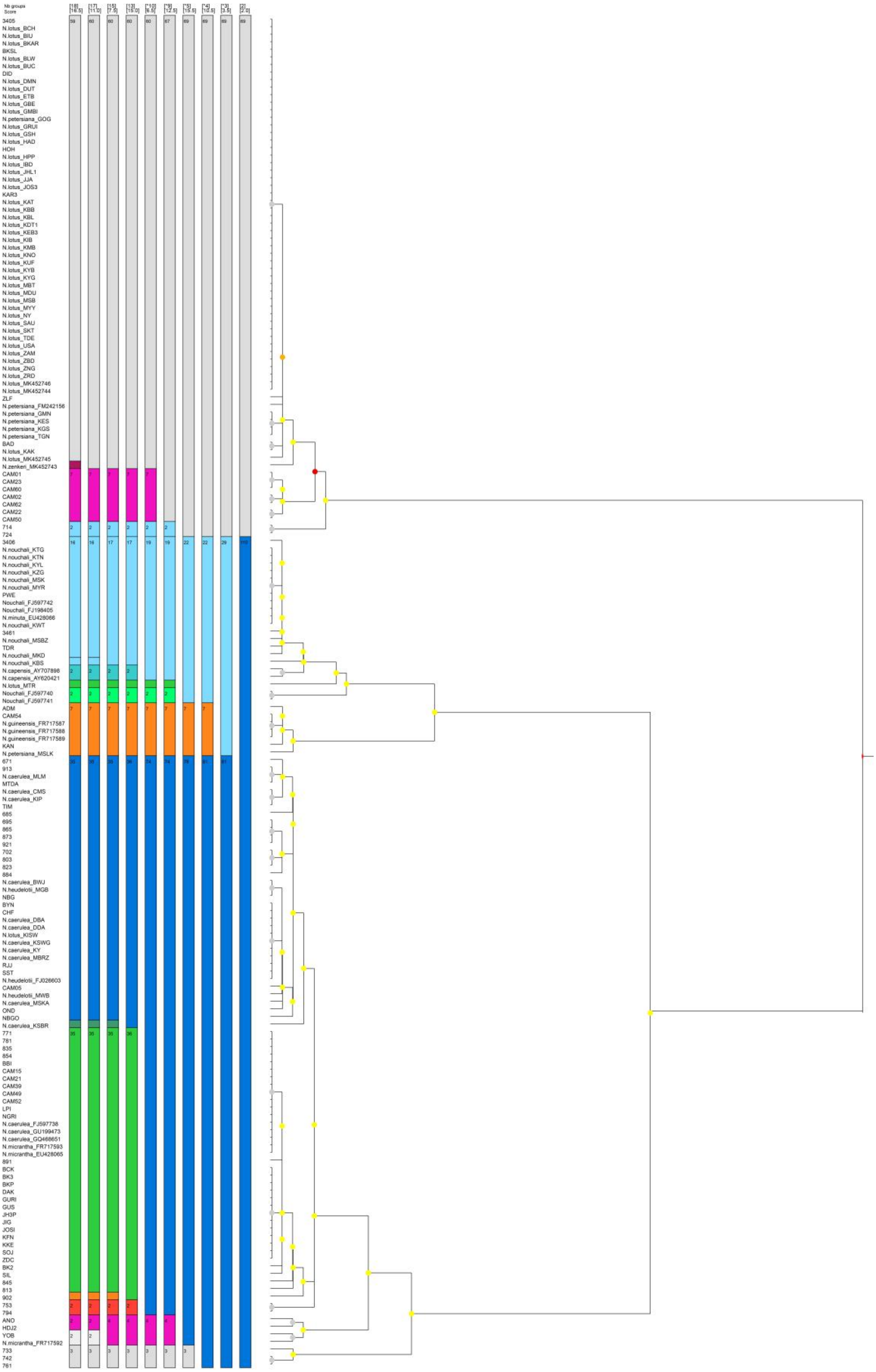

Figure S1 ITS partition strategy through ASAP algorithm.

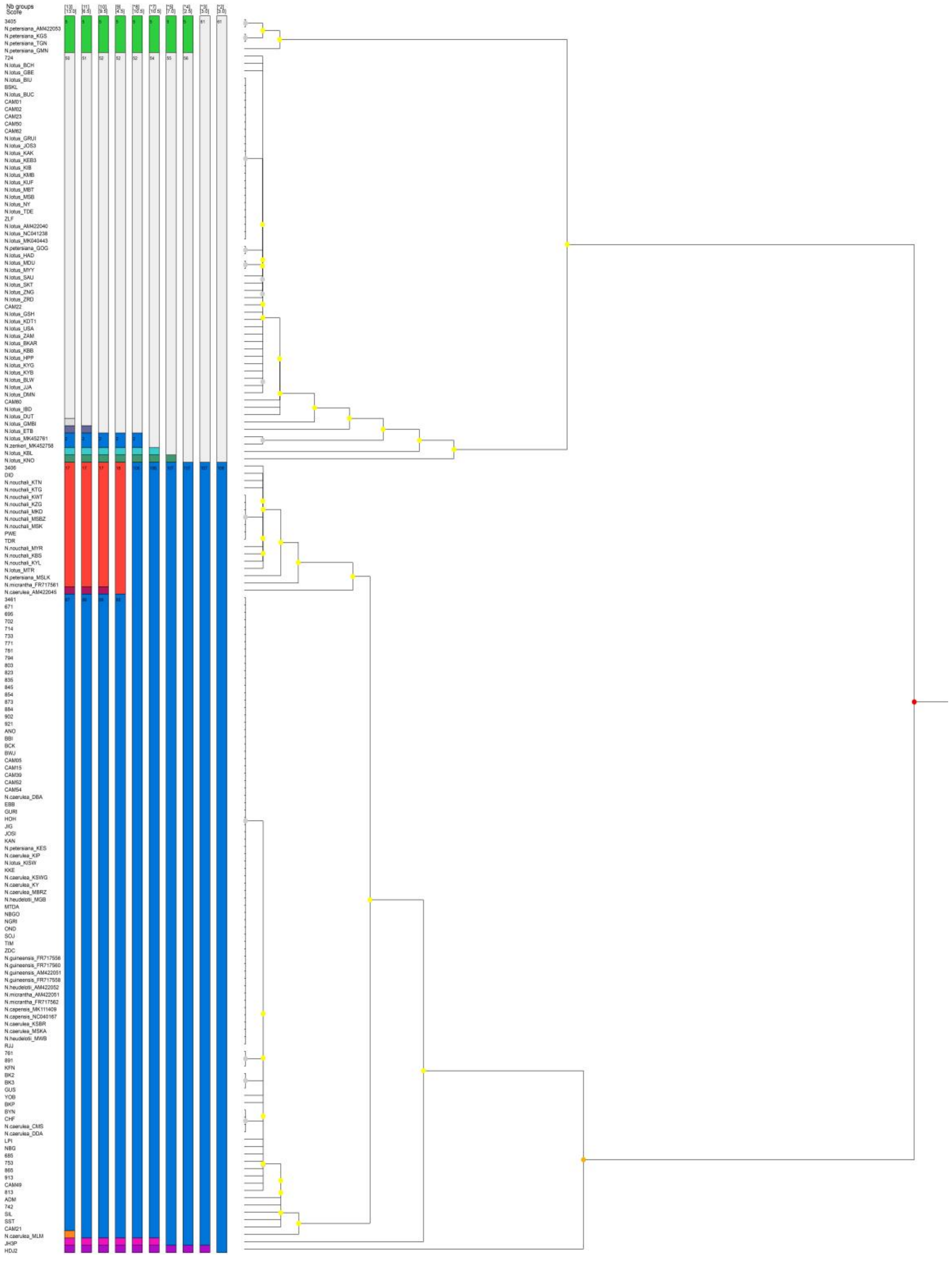

Figure S2 *trnT-trnF* partition strategy through ASAP algorithm.

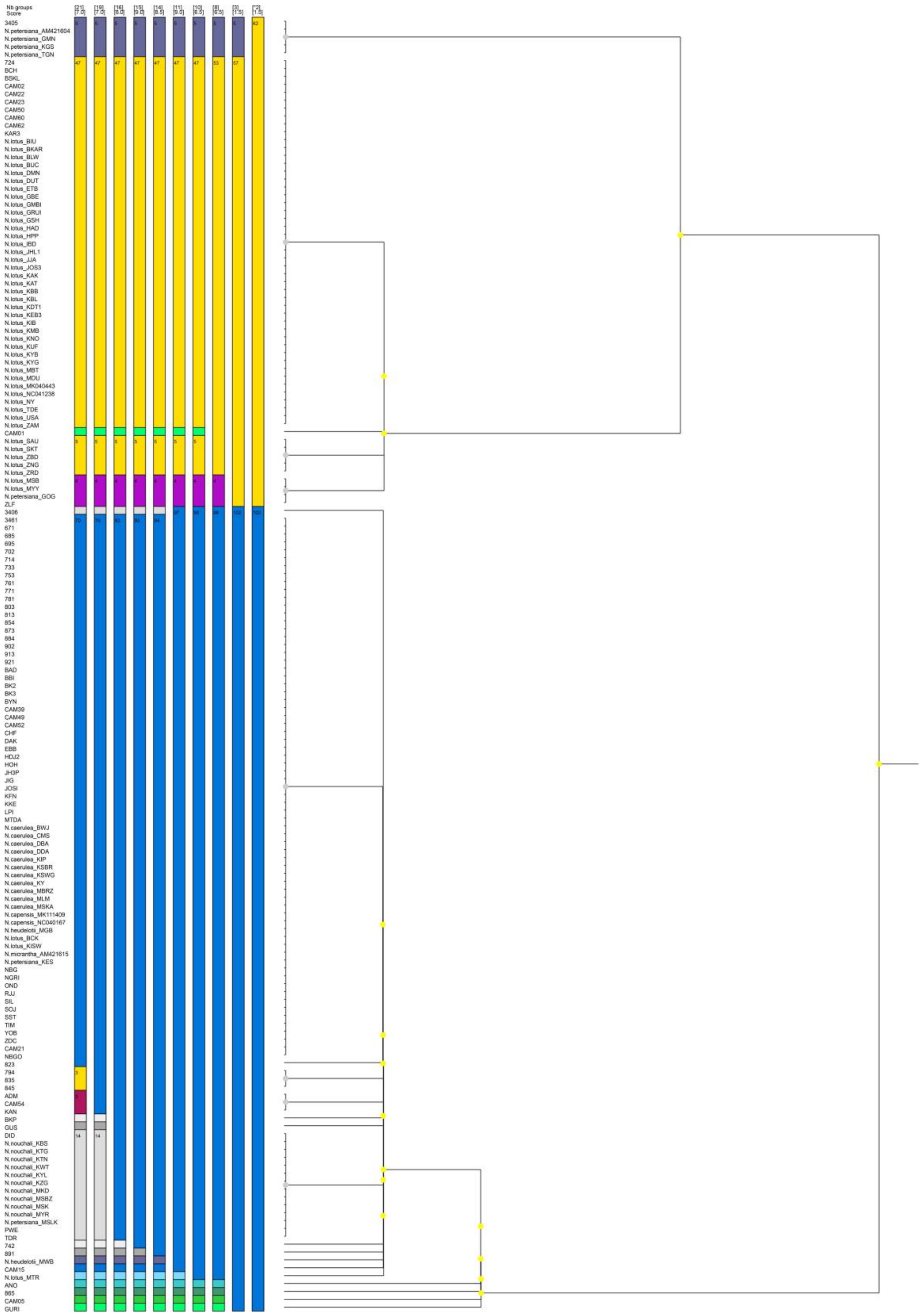

Figure S3 *rpl16* partition strategy through ASAP algorithm.

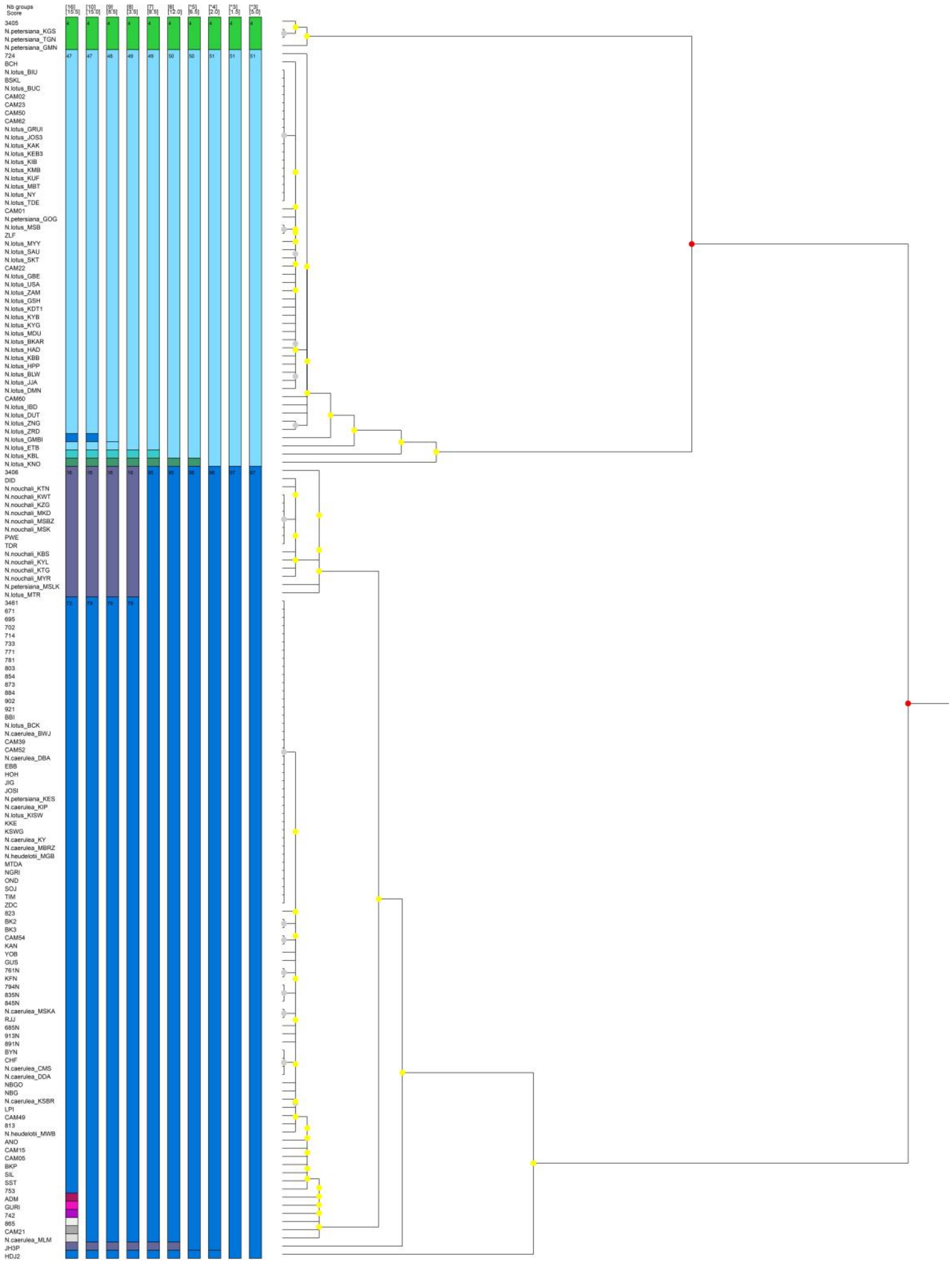

Figure S4 *trnT-trnF+rpl16* partition strategy through ASAP algorithm.

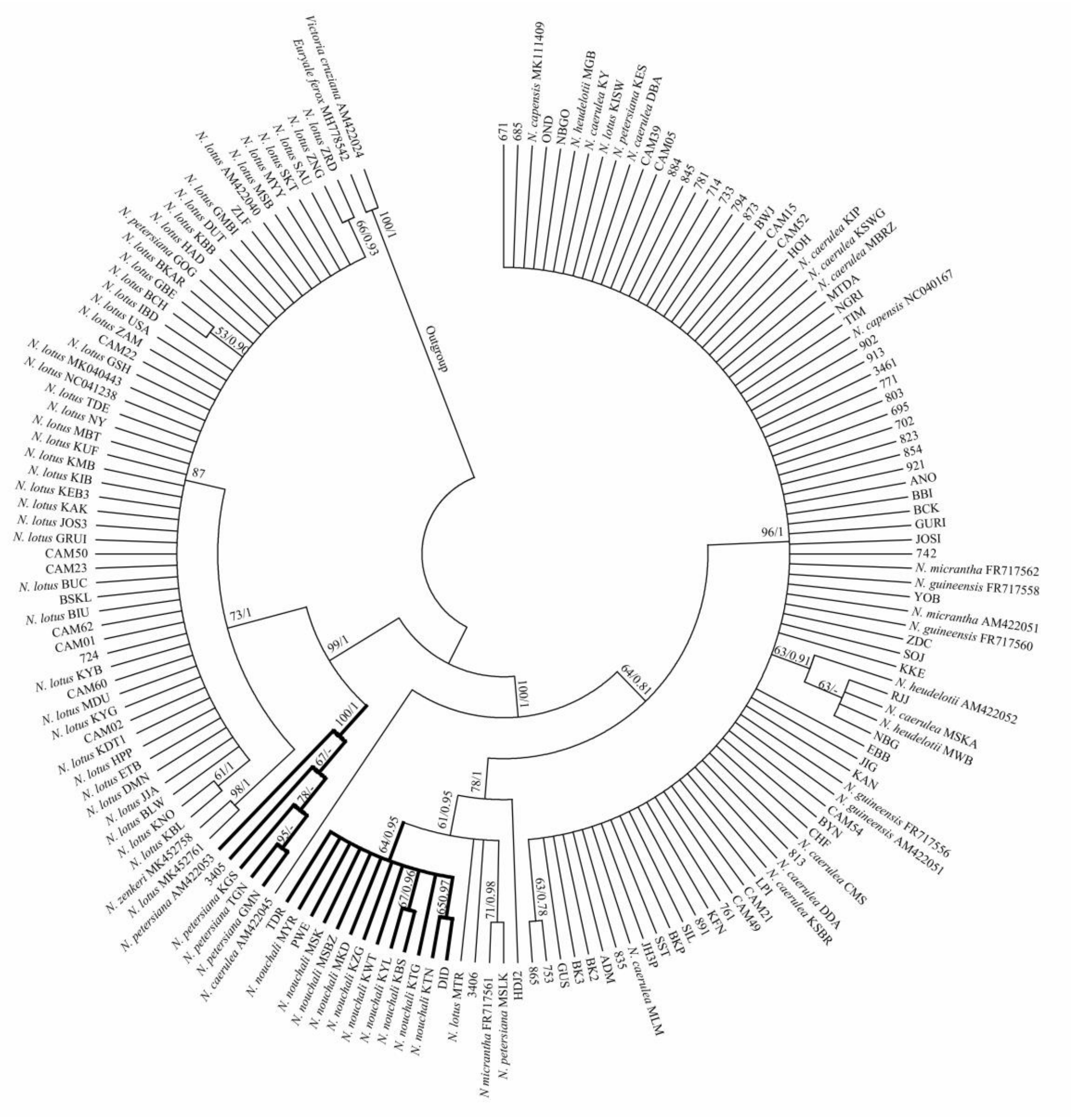

Figure S5 Phylogenetic tree constructed based on 167 *trnT-trnF* sequences of *Nymphaea.* Bootstrap values (> 50) and posterior probabilities (>0.5) based on NJ and BI analysis are shown above the branches. The rough branches mean identified species by *trnT-trnF* tree.

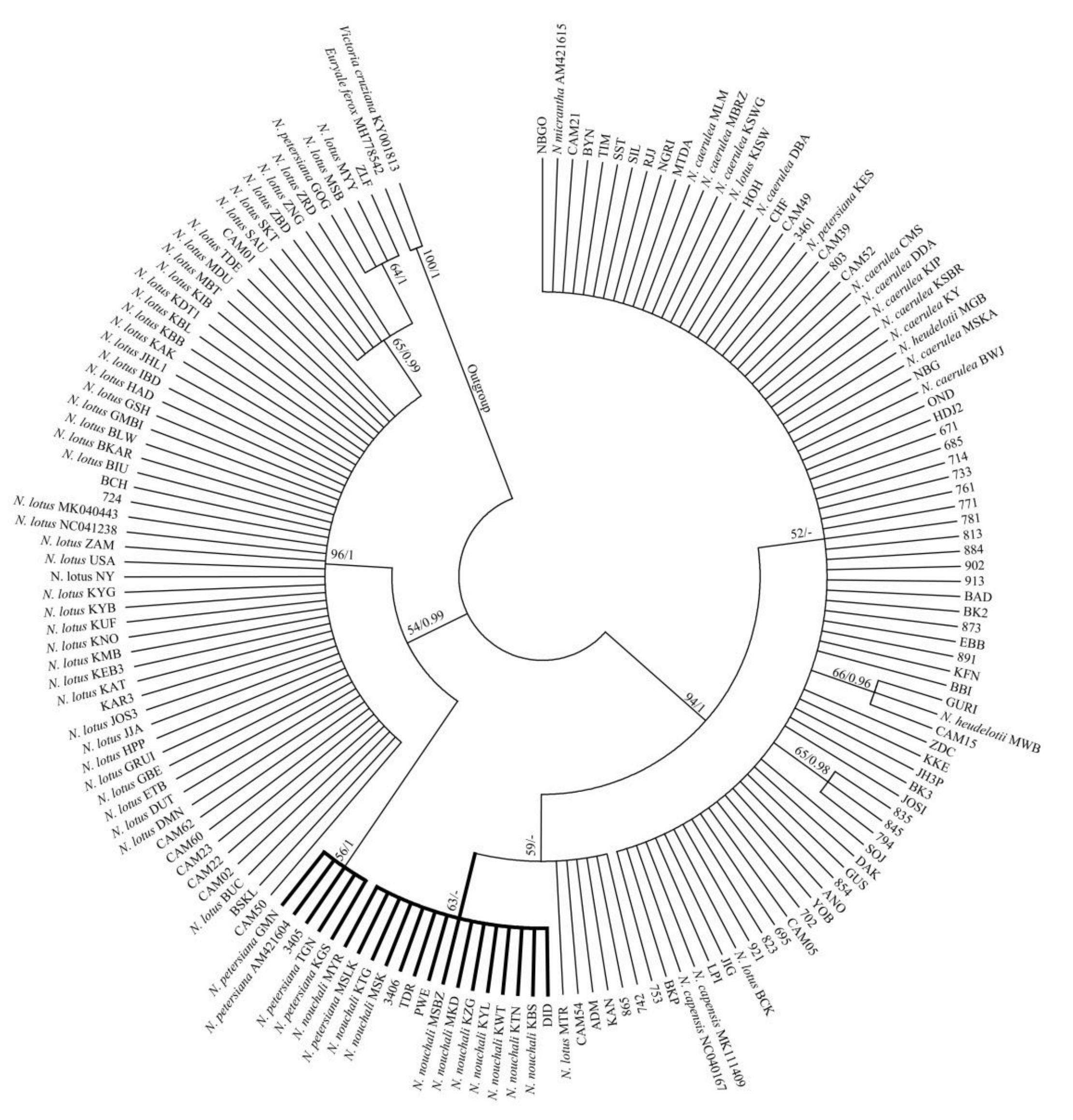

Figure S6 Phylogenetic tree constructed based on 164 *rpl16* sequences of *Nymphaea.* Bootstrap values (> 50) and posterior probabilities (>0.5) based on NJ and BI analysis are shown above the branches. The rough branches mean identified species by *rpl16* tree.

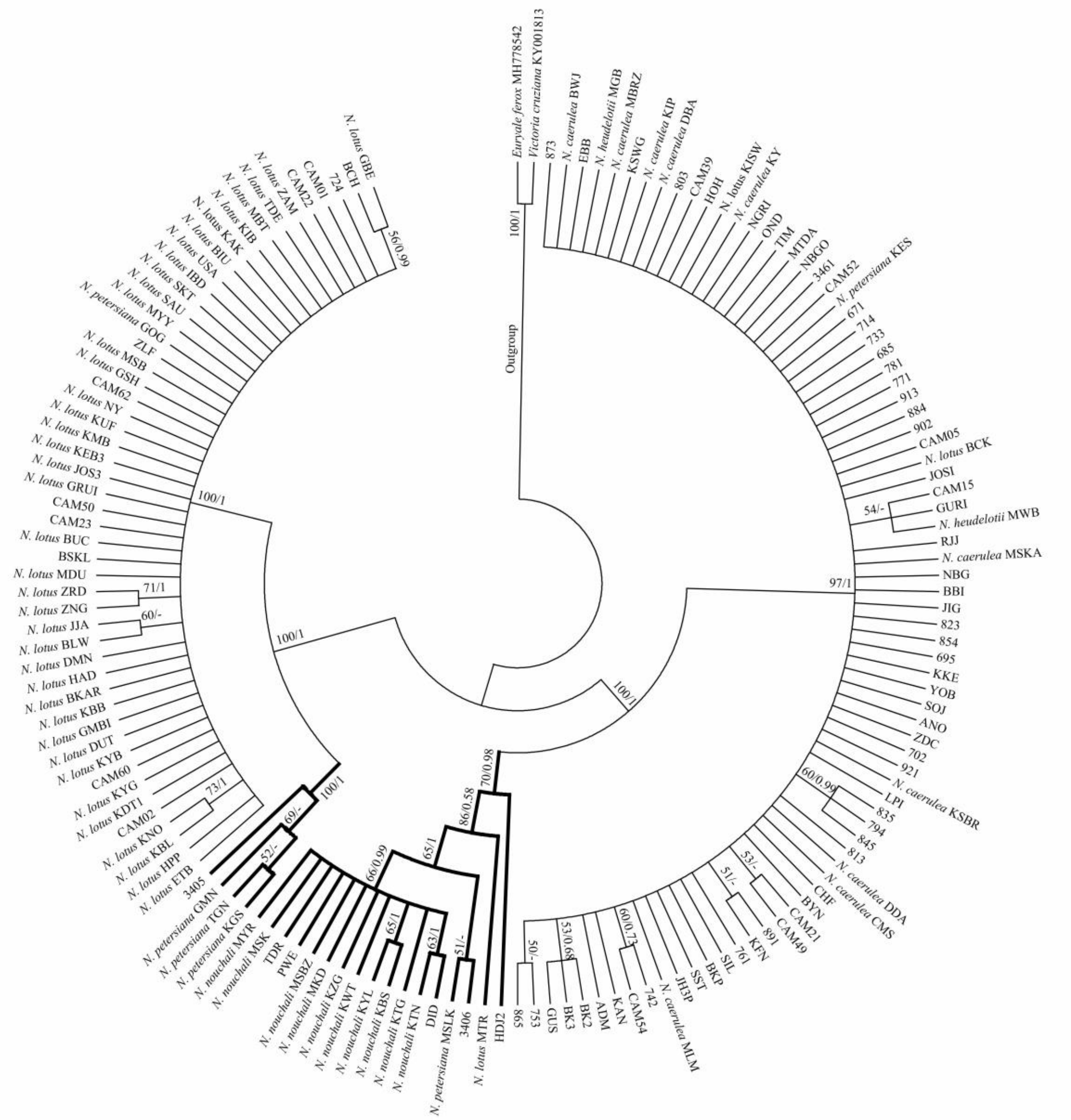

Figure S7 Phylogenetic tree constructed based on 152 *trnT-trnF+rpl16* sequences of *Nymphaea.* Bootstrap values (> 50) and posterior probabilities (>0.5) based on NJ and BI analysis are shown above the branches. The rough branches mean identified species by *trnT-trnF+rpl16* tree.
